# Metagenome-assembled genomes enhance bacterial read decontamination and variant calling in oral samples

**DOI:** 10.1016/j.isci.2025.113772

**Published:** 2025-10-14

**Authors:** Zunu An, Jun Hyung Cha, Kyu Ha Lee, Insuk Lee

**Affiliations:** 1 Department of Biotechnology, College of Life Science and Biotechnology, Yonsei University, Seoul, Republic of Korea; 2 Department of Epidemiology, Harvard T.H. Chan School of Public Health, Boston, MA 02115, USA; 3 Department of Nutrition, Harvard T.H. Chan School of Public Health, Boston, MA 02115, USA; 4 Department of Biostatistics, Harvard T.H. Chan School of Public Health, Boston, MA 02115, USA; 5 DECODE BIOME Co., Ltd., Incheon 21983, Republic of Korea

**Keywords:** Computational bioinformatics, Genomic analysis, Genomics

## Abstract

Whole genome sequencing (WGS) offers advantages over DNA chip-based genotyping, typically using blood-derived DNA. However, saliva and buccal samples—popular in direct-to-consumer tests—suffer reduced accuracy because of oral bacterial contamination. Decontamination strategies using decoy bacterial genomes yielded limited improvements, likely because they cover only a subset of oral bacteria with available isolate genomes. To overcome this, we developed a decontamination pipeline leveraging metagenome-assembled genomes (MAGs). Concordance analysis of variant calling between blood and matched oral samples confirmed the superiority of MAG-augmented decontamination over conventional methods relying mainly on isolate genomes. Although the underlying mechanism remains unclear, it particularly improves variant calls in GC-rich regions, recovering many likely pathogenic variants. Additionally, we demonstrate that certain bacterial genomic regions mimic human regions with clinically relevant variants, potentially confounding genotyping. These results highlight the need for MAG-based bacterial read decontamination to achieve accurate personal genotyping from non-invasive, self-collected oral samples.

## Highlights

• Decontamination of oral DNA samples via oral bacterial genomes improves genotyping
• Metagenome-assembled genomes enhanced bacterial read decontamination and genotyping
• Decontamination improves the detection of variants in challenging genomic regions
• Multiple oral bacteria harbor sequences that misalign to clinically relevant genomic regions

## Graphical abstract

**Figure:**
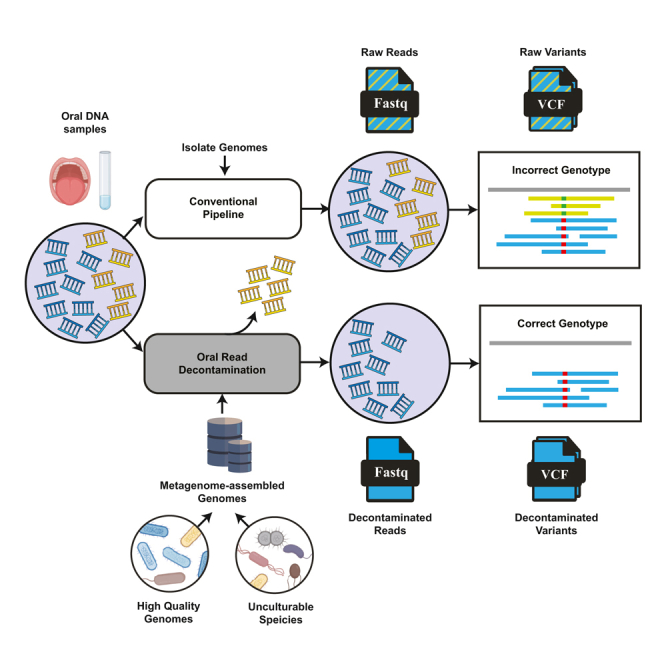

## Introduction

Whole genome sequencing (WGS) has become one of the most powerful tools in clinical genomics, enabling the comprehensive detection of a wide range of genetic variations, including single nucleotide polymorphisms (SNPs), small insertions and deletions (Indels), and copy number variations (CNVs). The quality of the DNA sample used can significantly impact genotyping accuracy. The Medical Genome Initiative, in its clinical WGS practice recommendations, designates genomic DNA (gDNA) extracted from whole blood as the gold standard, although saliva and tissue samples are also considered acceptable.^1^ However, saliva and buccal samples are often preferred in clinical settings that require non-invasive, self-collected samples, such as direct-to-consumer (DTC) genotyping services.

Previous studies have reported that variant detection from oral samples can achieve accuracy and reliability comparable to, though slightly lower than, that of blood samples.^2^^,^^3^^,^^4^ Instances of microbial reads misaligning to the human reference genome were identified,^5^^,^^6^ potentially compromising the accuracy of variant detection—particularly in regions with low coverage depth.^7^^,^^8^ Given that the human oral microbiome represents the second largest^9^ and among the most diverse microbial communities in the body,^10^ these findings highlight the risk of microbial contaminants interfering with genotyping accuracy.

Several approaches have been proposed to address microbial contamination in saliva samples. A common method involves appending bacterial decoy sequences to the human reference genome and aligning reads to filter out contaminants. While the previous decoy-based approach reduced the number of unmapped reads, it did not significantly improve variant calling accuracy.^6^^,^^11^ This limitation may stem from the low coverage of bacterial decoys, which are typically derived from NCBI RefSeq^12^ bacterial genomes that represent only a subset of the oral microbiota. Other studies have used the Human Oral Microbiome Database (HOMD)^13^ to analyze unmapped bacterial reads.^3^^,^^4^ However, HOMD is primarily based on isolated genomes from cultivated oral bacteria. As most oral microbial species remain uncultured, HOMD is likely to miss reads originating from these species. Therefore, an improved bacterial read decontamination method is needed to enhance clinical-grade genotyping from non-invasive, self-collected oral samples.

Recent advancements in genome-resolved metagenomics have enabled the culture-independent reconstruction of high-quality microbial genomes from metagenomic sequencing samples.^14^ These metagenome-assembled genomes (MAGs) substantially expand the genomic catalog of oral bacteria, enhancing the detection of bacterial contaminant DNA in saliva and buccal samples. However, the benefits of a “MAG-augmented” oral bacterial genome database for decontamination and variant calling remain unexplored.

In this study, we assess the impact of bacterial read decontamination on variant calling using the Human Reference Oral Microbiome (HROM),^15^ a MAG-augmented genomic catalog. We chose to use an oral-specific genome database rather than general databases such as GTDB^16^ to ensure high specificity to the oral niche and to minimize false positives that could arise from non-oral MAGs. Additionally, general genome databases are often prohibitively large for routine computational tasks, limiting their practicality in decontamination workflows. Our analysis demonstrates that employing a comprehensive MAG catalog improves bacterial read decontamination compared with conventional methods relying mainly on isolate genomes. Notably, HROM-based decontamination significantly improves the recovery of true variants in difficult-to-map regions. Furthermore, we identified numerous oral bacterial species with sequences matching clinically relevant variant regions, including pathogenic variants, many of which lack isolate genome references. These findings underscore the critical role of an HROM-based decontamination approach in ensuring accurate personal genotyping from oral samples.

## Results

### Metagenome-assembled genomes-augmented strategy uncovers hidden bacterial contaminants in oral samples

**Figure 1:**
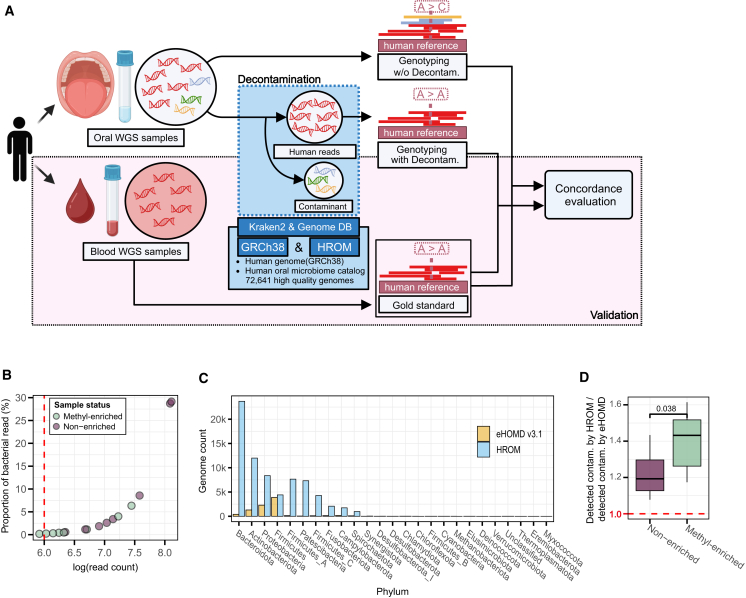
HROM-based decontamination of bacterial reads (A) An overview of the study design. The blue panel illustrates the decontamination step using Kraken2 and a custom genome database consisting of the human genome (GRCh38) and HROM. Human reads are shown in red, and contaminant reads are shown in other colors (blue, green, yellow). The red panel depicts the validation scheme, where genotyping results from decontaminated oral WGS samples are compared to those from blood WGS samples for concordance. (B) Scatterplot showing total contaminant reads per oral WGS sample, color-coded by methyl-enrichment status. The x axis shows log-scaled read count; y axis indicates the proportion of bacterial contaminants. The red dotted line at log(read count) = 6 indicates the threshold corresponding to 10^6^ microbial contaminant reads. (C) The bar plot compares the number of genomes per phylum between eHOMD (orange) and HROM (blue). (D) The boxplot displays the ratios of detected contaminant reads using HROM compared to eHOMD, for both non-enriched and methyl-enriched samples. The red dotted line at 1.0 represents the baseline where both databases detect an equal number of contaminant reads. A two-tailed Mann-Whitney U test was used to evaluate statistical significance. Boxplot elements: median (center line), interquartile range (box edges at 25th and 75th percentiles), and whiskers extending to 1.5× the interquartile range.

To detect and remove contaminated bacterial reads, we developed a streamlined decontamination approach within a widely used DeepVariant-based genotyping pipeline (Figure 1A). Our method employs the k-*mer*-based read classifier Kraken2^17^ and bacterial genomes from HROM. We constructed a custom database containing 72,641 high-quality bacterial genomes alongside the human genome (GRCh38^18^). Blood samples were collected from the Personal Genome Project Canada (PGPC),^19^ and matched oral samples (saliva or buccal swabs) were obtained in a follow-up study,^5^ with or without methylated DNA enrichment. A total of 20 samples from four individuals were analyzed.

Using HROM, we found that, on average, 4.95% of sequence reads in the oral-derived WGS samples originated from bacteria. All samples, except one, contained more than 10^6^ microbial contaminant reads, irrespective of methylated DNA enrichment during sequencing (Figure 1B). As expected, matched blood-derived WGS samples contained only background-level contaminant reads, averaging 0.38% (Table S1). The sample with the highest bacterial read contamination contained over 128 million (128,852,484) contaminant reads, accounting for 29.13% of the total reads. *Firmicutes, Actinobacteriota,* and *Bacteroidota* were the dominant phyla, exhibiting the highest relative abundance among the contaminated bacterial reads (Figure S1A).

Some previous studies have used HOMD^13^ for the analysis of contaminated bacterial reads.^3^^,^^4^ To compare the capability of bacterial read detection between HROM and the conventional database, HOMD, we used eHOMD (v3.1),^20^ the most recent version of the database available at the time of analysis. Since HROM is significantly larger (72,641 genomes, 3,426 species) and taxonomically more diverse across most phyla compared to eHOMD (8,622 genomes, 569 species), it likely provides enhanced sensitivity in contaminant detection (Figure 1C). Notably, the ratio of contaminant reads detected by HROM relative to eHOMD exceeds 1 and is higher in methyl-enriched (host DNA–enriched) samples than in non-enriched ones, indicating that the advantage of HROM over eHOMD in detecting contaminant bacterial reads is particularly pronounced in host DNA–enriched samples. (Figure 1D). This result may be explained by the fact that host DNA–enriched samples contain fewer bacterial reads overall, making contaminant detection more challenging. In such cases, the more comprehensive genome coverage provided by HROM likely enables higher recall in detecting residual contaminants compared to eHOMD. While some samples showed minimal differences in removal rates between HROM and eHOMD (<1%), others showed differences of up to 8% (Figure S1B). These results illustrate that significant bacterial contaminants remain undetectable in oral samples when using conventional bacterial genome databases that are primarily based on isolate genomes, even in host DNA enriched samples.

### Metagenome-assembled genomes-augmented bacterial read decontamination improves variant calling

To evaluate the impact of bacterial read decontamination on variant calling from oral samples, we first established baseline genotyping results obtained by aligning matched blood and oral-derived DNA reads to the GRCh38 human reference genome without applying any decontamination. On average, 99.97% of reads from blood samples and 95.42% from oral samples were successfully mapped. We then performed variant calling for each sample using DeepVariant,^21^ a method widely recognized for its high accuracy in benchmarking studies.^22^^,^^23^^,^^24^ After quality filtering, we identified an average of 3,868,159 (±18,271) SNPs and 977,135 (±13,307) Indels per sample. Compared to the previous study that used GATK,^22^ DeepVariant exhibited a substantial increase in detected variants, particularly for Indels, with an average of 750,756 additional Indels called (Table S2).

To systematically assess the effect of decontamination, we categorized all variants based on their type and minor allele frequency (MAF) into four groups: common SNPs (MAF ≥0.05), common Indels (MAF ≥0.05), rare SNPs (MAF <0.05), and rare Indels (MAF <0.05). Baseline evaluation metrics were established by assessing concordance between blood and raw oral samples, where no decontamination was applied. Variants identified from blood samples were regarded as the true set of variants, and those from oral samples were compared against this reference. To quantify performance, we calculated three evaluation metrics: precision, recall, and F1-score. Precision measures accuracy as the proportion of variants detected in both blood and oral samples out of all variants identified in oral samples. Recall measures sensitivity as the proportion of variants detected in both blood and oral samples out of all true variants present in blood samples. The F1-score, the harmonic mean of precision and recall, provides a balanced summary of both metrics. We then compared these baseline results to those obtained after HROM-based decontamination using the same evaluation metrics.

**Figure 2:**
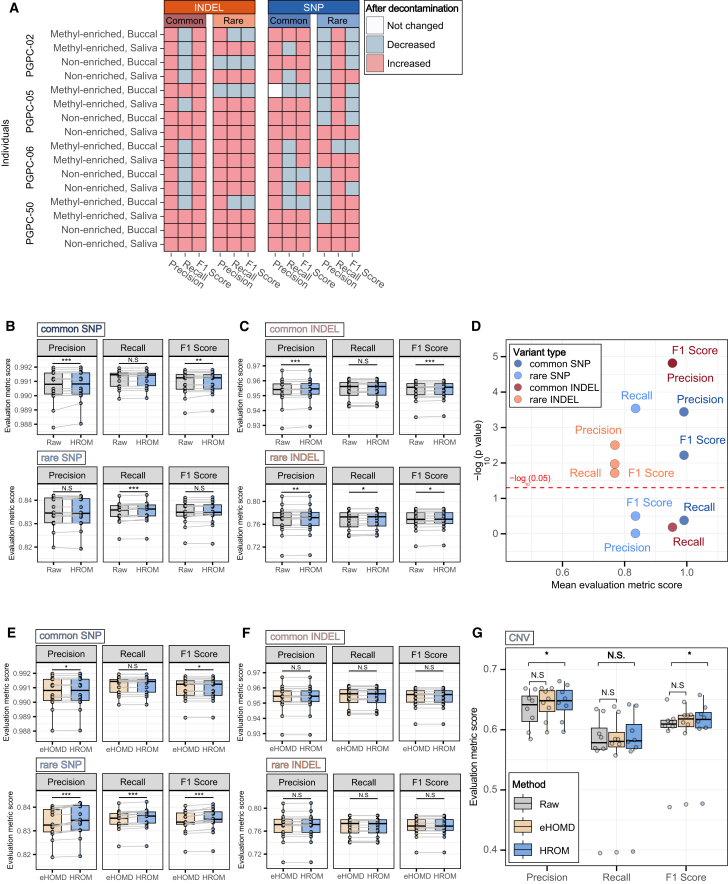
Decontamination of bacterial reads enhances overall variant calling (A) The heatmap illustrates changes in precision, recall, and F1-score for each variant type after decontamination. Each box represents the change in metrics, with red indicating an increase and blue indicating a decrease. Each row corresponds to a different oral sample, grouped in the following order: individuals, methylation status, and sample source. (B–C) Boxplots show the distribution of precision, recall, and F1 scores for common (MAF ≥0.05) and rare (MAF <0.05) SNPs (B) and indels (C), comparing HROM-based decontamination (light blue) with raw sequencing samples (light gray). Wilcoxon signed-rank test was used for comparison. ∗, *p* < 0.05; ∗∗, *p* < 0.01; ∗∗∗, *p* < 0.001. (D) The scatterplot displays the mean concordance evaluation metric score (x axis) and log-scaled *p*-value from a one-tailed Wilcoxon signed-rank test (y axis), comparing raw and decontaminated oral samples, with points colored by variant type. The red dotted line at −log(p) = 1.3, corresponding to a significance threshold of *p* = 0.05. (E–F) Boxplots show the distribution of precision, recall, and F1 scores for common (MAF ≥0.05) and rare (MAF <0.05) SNPs (E) and indels (F), comparing HROM-based decontamination (light blue) with the eHOMD-based decontamination of sequencing samples (light orange). Wilcoxon signed-rank test was used for comparison. ∗, *p* < 0.05; ∗∗∗, *p* < 0.001. (G) The boxplot displays the concordance evaluation metrics for CNVs across different decontamination statuses. One-tailed Wilcoxon signed-rank test was used to evaluate statistical significance. Boxplot elements consist of the median (center line), interquartile range (box edges at 25th and 75th percentiles), and whiskers extending to 1.5× the interquartile range. Wilcoxon signed-rank test was used for comparison. ∗*p* < 0.05.

Our analysis revealed that HROM-based decontamination significantly improved variant calling in most samples. Specifically, 12 out of 16 samples exhibited improvements in at least three of the four variant categories (Figure 2A). When evaluating aggregate SNP calling performance, HROM-based decontamination showed significant gains over raw sequencing data in 3 of 6 metrics (Figure 2B), and for indels, improvements were observed in 5 of 6 metrics (Figure 2C). For common SNPs as well as common and rare indels, bacterial read removal consistently enhanced precision and F1-scores, though this was not the case for rare SNPs. Evaluation metric values varied substantially across variant types, with performance generally descending in the order of common SNPs, common indels, rare SNPs, and rare indels. Although HROM-based decontamination yielded statistically significant improvements, the effect sizes were not always visually apparent in boxplots. To better capture these differences, we summarized the results using negative log-scaled *p*-values, which highlight significant improvements even when effect sizes were subtle (Figure 2D).

We further compared the effectiveness of HROM-based decontamination with eHOMD-based decontamination for variant calling. HROM-based decontamination outperformed eHOMD-based decontamination across most metrics (5 of 6) for both rare and common SNPs (Figure 2E), whereas no significant differences were observed for indels (Figure 2F). While HROM-based decontamination provided statistically significant improvements in SNP calling accuracy, eHOMD-based decontamination also improved performance relative to raw sequencing data, particularly for common SNPs (Figure S2A) and for both common and rare indels (Figure S2B). These results indicate that although isolate-based databases can enhance genotyping accuracy to some extent, MAGs provide additional coverage of contaminant reads that may be missed when using isolate genomes alone.

To further understand the contribution of MAG content in HROM, we performed an additional comparative analysis using a reduced version of HROM containing only isolate genomes. The results of isolate-only HROM-based decontamination show that the entire HROM-based decontamination performs significantly better in all metrics in rare Indels (Figure S2C). There was no significant difference between isolate-only and entire HROM-based decontamination in any of the metrics in the other stratifications (common Indel, common SNP, rare SNP). This is analogous to the original observation that isolated genomes by themselves perform reasonably well, as shown by eHOMD, but MAG augmentation provides additional benefits.

To assess the impact of decontamination on CNV calling, we utilized Illumina Canvas,^25^ a coverage-based CNV caller. Methyl-enriched samples were excluded from this analysis, as targeted sequencing methods introduce coverage biases that can interfere with CNV detection. When comparing the CNV concordance of HROM-based and eHOMD-based bacterial read decontamination to the baseline, we found that HROM-based decontamination significantly improved CNV concordance in precision and F1-scores, whereas eHOMD-based decontamination did not (Figure 2G). On average, HROM-based decontamination led to overall improvements in all metrics, with F1-score increasing by 1.24%, precision by 1.82%, and recall by 0.70%. These results further emphasize the importance of employing a MAG-augmented bacterial genome catalog to achieve effective bacterial read decontamination. Given that HROM demonstrated improved bacterial read decontamination compared to eHOMD in both SNP and CNV analyses, we focused the remainder of our study on HROM-based decontamination.

### Human reference oral microbiome-based decontamination particularly improves variant calling in difficult-to-map regions

To evaluate the characteristics of variants recovered through our decontamination pipeline, we analyzed the distribution of true variants that were either recovered or lost after decontamination. These variants, detected exclusively in both blood and saliva samples post-decontamination, were categorized based on the difficulty of read alignment. The Genome in a Bottle (GIAB) Consortium defines difficult regions of the human genome—such as tandem repeats, homopolymers, or segmental duplications—where alignment accuracy is reduced and variant calling is challenging.^26^

**Figure 3:**
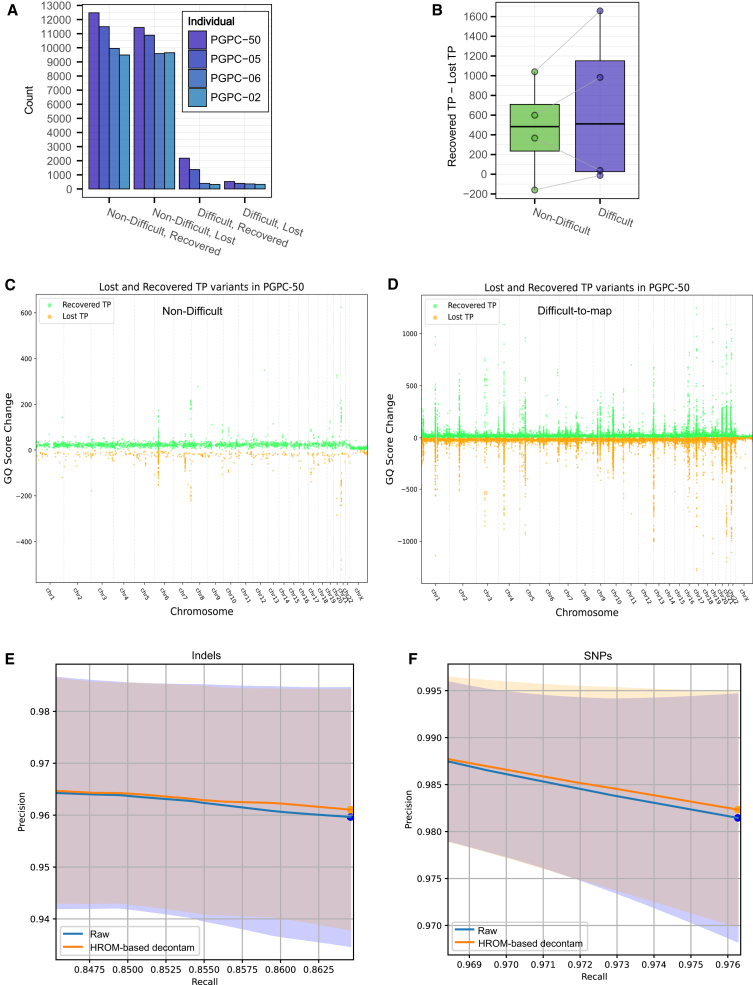
Decontamination of bacterial reads enhances recovery of variants particularly in difficult-to-map genomic regions (A) The bar plot represents number of variants that were recovered or lost after decontamination per individual, stratified by difficult and non-difficult regions. (B) The boxplot shows the difference in variant counts between recovered and lost variants, stratified by the difficult/non-difficult region. Boxplot elements: median (center line), interquartile range (box edges at 25th and 75th percentiles), and whiskers extending to 1.5× the interquartile range. (C–D) Both scatterplots display each recovered variant (green) and lost variant (orange) according to its genomic position (x axis) and genotype quality score change (y axis). While (C) shows variants in non-difficult regions, (D) shows variants in difficult regions. (E–F) Both mean precision-recall curves comparing two different methods, HROM-based decontamination (orange) and raw (blue) in high-GC promoters, stratified by variant type. The shaded regions indicate sample variability of one standard deviation, where overlap between the two methods is shown in gray. While (E) shows precision-recall metrics of Indels, (F) shows SNPs.

While the number of recovered true variants was only slightly higher (1.04%) than lost variants in non-difficult regions, recovered true variants in difficult-to-map regions outnumbered lost variants by 2.01-fold (Figures 3A and 3B). An increase in Genotype Quality (GQ) scores was observed for recovered variants, whereas a decrease was noted for lost variants. In the saliva sample of PGPC-50, a near-equivalent tradeoff between recovered and lost variants was observed in non-difficult regions (Figure 3C). However, in difficult-to-map regions, substantially more variants were recovered compared to those that were lost (Figure 3D).

To further investigate which specific difficult-to-map regions were most impacted by decontamination, we analyzed the precision of variant calling across different recall values in promoter regions, which are known to be challenging to sequence due to their high GC content.^27^ Our results showed that decontamination led to improved precision of variant calling across recall values for both indels and SNPs in these regions (Figures 3E and 3F). To validate whether these improvements were indeed occurring in high GC-content regions, we performed identical computations in genomic regions with GC content greater than 85%, as defined by the GIAB consortium.^26^ The precision of variant calling improved across all thresholds for indels, while most threshold values showed no difference for SNPs (Figures S3A and S3B). These results suggest that HROM-based bacterial read decontamination particularly improves variant calling in sequencing-resistant promoter regions and GC-rich genomic regions, although the underlying mechanism remains unclear.

### Metagenome-assembled genome-augmented decontamination recovers damaging missense mutations

**Figure 4:**
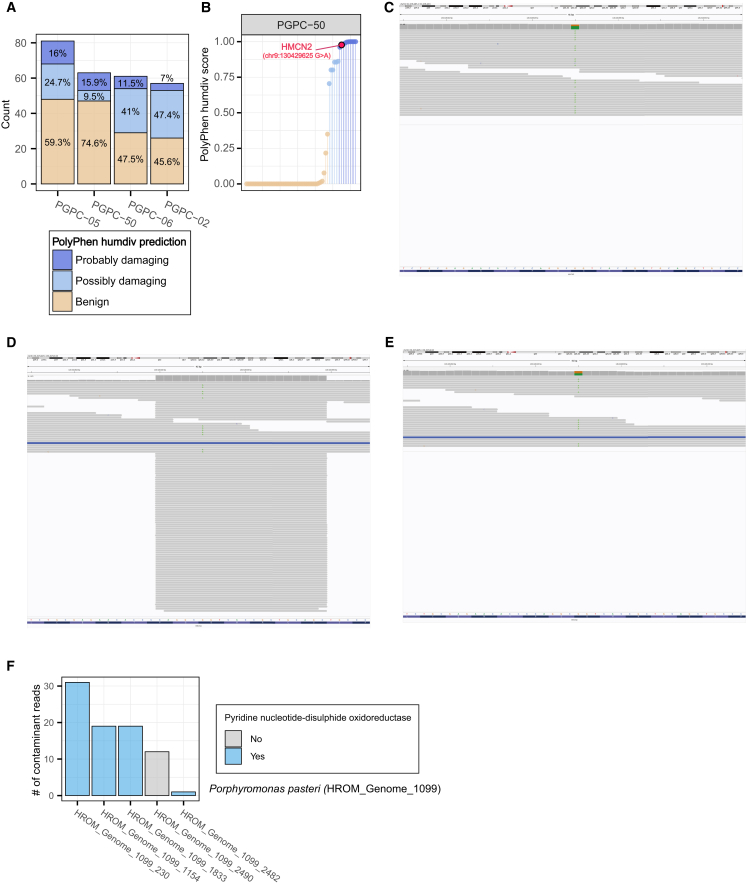
HROM-based bacterial read decontamination enables recovery of missense variants with clinical relevance (A) A stacked bar chart categorizing PolyPhen-2 HumDiv predictions of recovered, true-positive missense variants across four samples from PGPC-02 through PGPC-50. Variants are categorized as benign (yellow), possibly damaging (light blue), and probably damaging (dark blue), with percentages indicating the proportion of each category within each sample. (B) Recovered missense variants of PGPC-50, sorted by ascending PolyPhen-2 HumDiv scores. Variants are color coded according to their predicted functional effect of benign (yellow), possibly damaging (light blue), and probably damaging (dark blue). (C–E) Integrated genomics viewer (IGV) visualization of sequencing read pileups. The vertical gray bars on the top indicate coverage depth per position. (C) shows a heterozygous variant call, highlighted with an orange/green marker, in the HMCN2 gene in the blood sample of PGPC-50. (D) shows a falsely uncalled SNP of HMCN2 in the saliva sample of PGPC-50. (E) shows the identical saliva sample decontaminated to recover the correct heterozygous call, as well as a uniform coverage depth. (F) A bar plot shows the distribution of contaminant reads aligned to HMCN2 (chr9:130429625) when mapped to the pangenome of *P. pasteri* (HROM_Genome_1099). The x axis shows the five genes where reads aligned, with colors indicating pyridine nucleotide-disulphide oxidoreductase annotation.

Given that HROM-based bacterial read decontamination recovered many true variants, we next investigated whether these included pathogenic variants. If so, our decontamination pipeline could have important clinical implications for individuals undergoing genotyping for preventive medicine. To assess the clinical relevance of the recovered true variants, we annotated them using PolyPhen-2,^28^ applying the more stringent HumDiv model to identify those predicted to be most damaging. A substantial proportion of recovered true missense variants had high PolyPhen HumDiv scores, with 30.64% classified as “possibly damaging” and 12.6% as “probably damaging” (Figure 4A). Notably, their recovery contributed to improved precision-recall metrics across the entire coding region (Figures S4A and S4B).

Among the recovered missense variants, a notable example was identified in the HMCN2 gene (Figures 4B and S4C), which encodes hemicentin-2, a key protein involved in extracellular matrix remodeling, essential for cell adhesion and tissue integrity.^29^ Previous studies suggest that HMCN2 may influence early-stage HIV-1 replication, indicating a potential role in viral pathogenesis.^30^ A heterozygous mutation in the protein-coding region of HMCN2 (chr9:130429625 G>A), predicted to be highly damaging (PolyPhen-2 score = 0.977), was called in the blood sample of PGPC-50 but remained uncalled in the corresponding saliva sample due to contamination. To investigate the effect of contaminant reads on the masking of the HMCN2 variant, we examined read pileup images. In the blood sample, the heterozygous variant was clearly supported by reads carrying both the reference and alternate alleles (Figure 4C). In contrast, the corresponding saliva sample exhibited a localized increase in read depth, obscuring the variant and preventing its detection (Figure 4D). Notably, after applying the decontamination pipeline to the saliva sample, the variant was successfully recovered while retaining human-origin reads, enabling the correct heterozygous genotype call (Figure 4E).

When we extracted these contaminant reads and reclassified them using HROM, we found that they were predominantly assigned to *Porphyromonas pasteri* (HROM_Genome_1099, 39/41 of paired-end reads), with the remaining reads classified within the order Bacteroidales (2/41 of paired-end reads). Upon aligning these reads to the non-redundant gene sequences of the *P. pasteri* pangenome, we observed that all reads mapped to five genes originating from five distinct oral *P. pasteri* strains. Notably, four of these five genes were annotated as pyridine nucleotide-disulfide oxidoreductases (Figure 4F), suggesting that bacterial reads confounding a specific variant call primarily originate from a specific gene in a specific bacterial species, rather than from diverse genomic regions. This finding aligns with previous reports indicating that bacterial reads can spuriously align to the human reference genome when insert lengths are very short (≤30 bp).^6^ Overall, our results highlight the efficacy of the decontamination pipeline in recovering potentially damaging variants by accurately distinguishing human-origin reads from bacterial contaminants.

### Oral bacterial genomic regions align to human regions with clinically relevant variants

Since publicly available blood-oral matched samples were limited to four individuals, our ability to generalize our findings was constrained. To mitigate this, we analyzed oral bacterial genomic regions with sequence similarity to human genomic regions containing clinically relevant variants from ClinVar.^31^ We hypothesized that homologous oral microbial sequences could obscure the detection of clinically verified ClinVar variants. This approach enabled us to evaluate the potential for genotyping interference across a broader range of oral bacterial genomic regions represented in HROM, extending beyond those observed in the individual samples analyzed.

**Figure 5:**
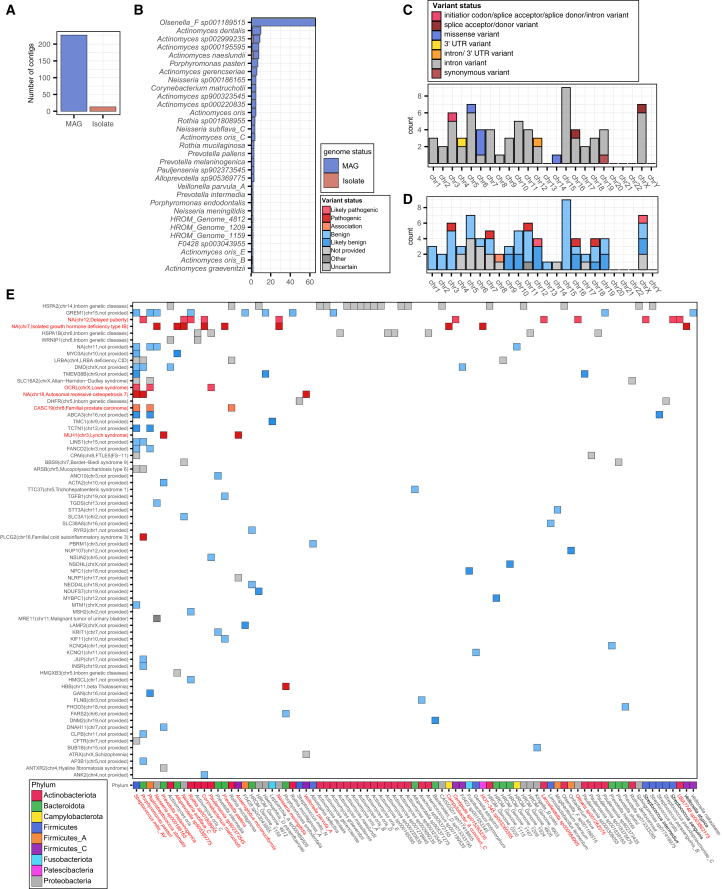
Oral microbial contigs homologous to the genomic regions containing ClinVar variants (A) Each bar represents the number of contigs from conspecific MAGs and isolate genomes of respective species that show high similarity to human genome regions containing clinical variants in ClinVar. (B) Number of contigs per species that have regions with high similarity to human genome regions containing clinical variants in ClinVar. Color denotes whether the contig is from an isolate strain or a MAG strain. (C) Bar plot shows the distribution of 82 ClinVar variant regions with high similarity to HROM, stratified by chromosome and colored by variant type/status. (D) Bar plot shows the distribution of 82 ClinVar clinical variants with high similarity to HROM, stratified by regions across human chromosomes, colored by pathogenicity of the variant. (E) Heatmap illustrates highly matched regions between bacterial species and clinical variant regions, with colors indicating variant pathogenicity. Diseases associated with ClinVar variant regions that share homologous sequences with multiple bacterial species, as well as bacterial species with homologous genomic regions linked to human diseases and lacking isolate genome references, are highlighted in red.

For this analysis, we extracted ClinVar variant positions along with their ±150 bp flanking sequences and searched for homologous bacterial sequences in HROM using the ultrafast aligner MMseqs2.^32^ We applied thresholds of ≥50% coverage and ≥80% sequence identity. Then, we simulated reads from the matched bacterial regions and aligned them to the flanking sequences, retrieving only those with successful alignments. This identified 270 of 72,641 bacterial genomes across 83 of 3,426 species in HROM with high similarity to 82 ClinVar variant regions. Notably, the majority of these highly matched regions originated from MAG contigs, highlighting their substantial impact on variant detection (Figure 5A). Among the identified species, *Olsenella_F sp001189515* harbored the highest number of contigs (Figure 5B) and conspecific genomes (Figure S5) with strong matches to ClinVar variant regions, suggesting that contamination from this species is most likely interfering with genotyping accuracy. This finding suggests that specific microbial taxa may significantly impact human clinical genetics. While most clinical variants in these matched regions were classified as intronic, some contained functionally significant variants, including splice donor/acceptor site mutations and missense variants in coding regions (Figures 5C and 5D).

Regarding pathogenicity, although most matched regions were classified as benign (69.51%, 57/82), pathogenic variants (ClinVar status: Likely pathogenic, Pathogenic, or Association) were distributed across multiple chromosomes. Some pathogenic variant regions had homologous sequences in multiple bacterial species and were associated with diseases such as isolated growth hormone deficiency type IB, delayed puberty, autosomal recessive osteopetrosis, Lowe syndrome, familial prostate carcinoma, and Lynch syndrome (Figure 5E). Notably, certain bacterial species with matched pathogenic variant regions had only MAGs available, with no corresponding oral isolate genomes: *Streptococcus mitis_AV, Porphyromonas pasteri, Peptostreptococcus stomatis, Neisseria* sp*000186165, Prevotella melaninogenica, Corynebacterium matruchotii, Rothia* sp*001808955, Neisseria subflava_C, Alloprevotella* sp*905369775, Prevotella pallens, Pauljensenia* sp*902373545, Veillonella parvula_A, Porphyromonas endodontalis, Neisseria meningitidis, Anaeroglobus micronuciformis, among others.* This finding suggests that interference from these bacterial species may lead to undetected genotyping errors for pathogenic variants, underscoring the necessity of an HROM-based approach.

## Discussion

In this study, we demonstrate the effectiveness of bacterial read decontamination in improving variant calling from WGS of oral-derived samples. By leveraging an oral bacterial genome database that includes previously uncharacterized uncultured species, we achieved higher variant-calling accuracy compared to conventional methods that rely primarily on isolate genomes. This approach enabled the recovery of numerous clinically relevant variants. Compared to conventional oral microbial genome databases such as eHOMD, HROM demonstrated the superior detection of bacterial contaminants, leading to improved variant-calling performance. One key factor contributing to HROM’s performance is the inclusion of high-quality MAGs, including only non-chimeric MAGs that meet stringent quality thresholds (≥90% completeness, <5% contamination). It should be noted that CheckM contamination reflects the proportion of redundant bacterial sequences within a MAG, rather than fragments of human DNA. This expanded the taxonomic representation of the oral microbiome, allowing HROM to capture microbial contaminants that are missed by conventional databases, particularly from phyla underrepresented in isolate collections.

Furthermore, our findings highlight that HROM-based bacterial read decontamination significantly improves recovery of true variants in difficult-to-map regions while maintaining a balanced tradeoff between recovered and lost variants in non-difficult regions, preserving overall accuracy. Notably, recovered variants were enriched in challenging regions such as promoters, particularly those with high GC content, which are prone to sequencing difficulties. The observed improvement in high GC-content regions across the genome suggests that bacterial misalignment in these regions may contribute to genotyping errors.

We also demonstrate that HROM-based bacterial read decontamination restores numerous clinically relevant variants in coding regions, many of which are predicted to be potentially damaging, including a variant in HMCN2. The identification of contaminant reads masking HMCN2 as originating from *Porphyromonas pasteri* underscores the necessity of leveraging an HROM-based microbial genome database to improve variant detection accuracy in human WGS samples.

While most samples showed improvement in genotyping accuracy, a few showed no improvement or even a decrease in accuracy. Although our conservative approach effectively removes potential microbial contaminants, it may also inadvertently remove true-positive human reads. Such losses can obscure genuine variants by generating homozygous reference calls or, conversely, introduce spurious variants through incorrect genotype assignments.

Finally, we demonstrated that bacterial genomic regions can exhibit high similarity to regions containing validated, clinically relevant variants from ClinVar, posing a potential risk of interfering with variant interpretation. Notably, certain bacterial species with matched pathogenic variant regions had only MAGs available, with no corresponding isolate genomes, highlighting the necessity of an HROM-based approach for detecting these variants. Our findings suggest that conventional pipelines relying mainly on isolated bacterial genomes may overlook substantial contamination from the oral microbiota, potentially compromising genotyping accuracy. This issue will become increasingly critical as DTC genotyping services transition from DNA chip-based to WGS-based approaches. These insights provide a foundation for developing a systematic decontamination pipeline for WGS-based genotyping of oral-derived gDNA samples, ultimately improving genotyping reliability and advancing genomic research.

### Limitations of the study

Our study has certain limitations. First, the relatively small dataset may limit the generalizability of our findings across diverse populations. Second, while HROM is one of the most comprehensive human oral microbial genome catalogs composed of high-quality genomes, its dataset is primarily derived from samples collected in the USA and China. This geographic bias may introduce regional differences in bacterial read decontamination. Additionally, while bacteria are the primary source of contamination, other potential contaminants, such as viruses and fungi, should also be considered for a more comprehensive decontamination strategy.

## Resource availability

### Lead contact

Requests for further information and resources should be directed to and will be fulfilled by the lead contact, Insuk Lee (insuklee@yonsei.ac.kr).

### Materials availability

The HROM database, including a description of the assembly pipeline, assembled genomes with associated metadata, is publicly available at www.decodebiome.org/HROM/.

### Data and code availability

• All sequencing data used in this study were obtained from publicly available datasets in the NCBI Sequence Read Archive (SRA) under the accession number SRA: PRJNA523344.
• No new sequencing data were generated in this study.
• All original code used in this study has been deposited in GitHub (https://github.com/netbiolab/OralDecontam) and is publicly available.
• Any additional information required to reanalyze the data reported in this article is available from the lead contact upon request.

## Acknowledgments

This research was supported by Korea Health Technology R&D Project, 10.13039/501100003710Korea Health Industry Development Institute (KHIDI), Ministry of Health & Welfare, Republic of Korea grant HI19C1344 (J.H.C.).

## Author contributions

Z.A., J.H.C., and I.L. conceived and designed the study. Z.A. and J.H.C. conducted analysis of metagenomic filtering followed by variant calling under supervision of K.H.L. and I.L. J.H.C., Z.A., and I.L. wrote and edited the article.

## Declaration of interests

The authors declare no competing interests.

## Declaration of generative AI and AI-assisted technologies in the writing process

The authors used ChatGPT during the preparation of this work to assist with language and readability improvements. All content generated with the tool was carefully reviewed and edited by the authors, who take full responsibility for the final version of the article.

## STAR★Methods

### Key resources table

**Table:** 

REAGENT or RESOURCE	SOURCE	IDENTIFIER
**Deposited data**		

Blood and oral WGS data	Reuter et al.^19^	SRA: PRJNA523344
GRCh38 reference genome (GCA_000001405.15), no alt analysis set	NCBI	https://ftp.ncbi.nlm.nih.gov/genomes/all/GCA/000/001/405/GCA_000001405.15_GRCh38/seqs_for_alignment_pipelines.ucsc_ids/
GIAB v3.0 stratification BED files	Olson et al.^26^	https://ftp-trace.ncbi.nlm.nih.gov/ReferenceSamples/giab/release/genome-stratifications/v3.0/GRCh38/

**Software and algorithms**		

fastp	Chen et al.^33^	https://github.com/OpenGene/fastp
BWA-MEM	Li and Durbin^34^	http://bio-bwa.sourceforge.net
SAMtools	Li et al.^35^	https://github.com/samtools/samtools
Picard	Broad Institute^36^	https://broadinstitute.github.io/picard/
Kraken2	Wood et al.^17^	https://ccb.jhu.edu/software/kraken2/
CheckM2	Chklovski et al.^37^	https://github.com/chklovski/CheckM2
GUNC	Orakov et al.^38^	https://github.com/grp-bork/gunc
DeepVariant	Poplin et al.^39^	https://github.com/google/deepvariant
BCFtools	Danecek et al.^40^	https://github.com/samtools/bcftools
hap.py	Krusche et al.^41^	https://github.com/Illumina/hap.py
Canvas	Roller et al.^25^	https://github.com/Illumina/canvas
Truvari	English et al.^42^	https://github.com/ACEnglish/truvari
Variant Effect Predictor	McLaren et al.^43^	https://github.com/Ensembl/ensembl-vep
Integrative Genomics Viewer	Thorvaldsdottir et al.^44^	https://igv.org
Prokka	Seemann^45^	https://github.com/tseemann/prokka
MMseqs2	Steinegger and Soding^32^	https://mmseqs.com
InterProScan	Jones et al.^46^	https://www.ebi.ac.uk/interpro/download/interproscan/
GATK4 Funcotator	GATK	https://gatk.broadinstitute.org/hc/en-us/articles/360057439472-Funcotator
FCS-GX	Astashyn et al.^47^	https://github.com/ncbi/fcs-gx
Python version 3.11	Python Software Foundation	https://www.python.org/
R version 4.4	R Foundation for Statistical Computing	https://www.r-project.org/
R package ‘ggplot2’	Wickham^48^	https://ggplot2.tidyverse.org
R package ‘ggsignif’	Ahlmann-Eltze et al.^49^	https://const-ae.github.io/ggsignif/

### Method details

#### Collection and preprocessing of WGS datasets

We downloaded blood and oral WGS datasets from the Sequence Read Archive (SRA)^50^ under the accession SRA: PRJNA523344. We collected total 16 paired blood-oral whole genome shotgun sequencing samples from four individuals (PGPC-02, PGPC-05, PGPC-06, and PGPC-50). General sequence alignment characteristics are outlined in previous research from which the data were obtained.^5^

Adapter trimming was performed with Fastp (v0.23.4)^33^ with recommended Phred quality filtering and length filtering options “-q 15 -l 15 –detect_adapter_for_pe”. Reads were aligned to the GRCh38^18^ reference genome (GCA_000001405.15_GRCh38) using BWA-MEM (v0.7.17).^34^ Metrics for BAM files were calculated using SAMtools (v1.20).^35^ PCR duplicates were identified and marked using Picard (v3.1.1).^36^

#### Bacterial read decontamination

HROM,^15^ a human oral microbiome catalog containing 3,426 prokaryotic species and 72,641 high-quality genomes, was used as the decontamination database. We constructed a custom Kraken2^17^ genome database comprising the human reference genome (vGRCh38) and 72,641 HROM genomes. Reads were mapped to this database using Kraken2 with the “--confidence 0.2” option. Only reads not classified as domain Bacteria or its subsequent lineages were retained for genotyping.

For comparison, we obtained oral microbial genomes from eHOMD (v3.1)^20^ and applied the same quality criteria as HROM. Genome completeness and contamination were assessed using CheckM2 (v1.0.1),^37^ retaining genomes with ≥ 90% completeness and ≤ 5% contamination. CheckM contamination represents redundant bacterial sequences within a MAG, not human DNA fragments. Genome chimerism was evaluated using Genome UNClutterer (GUNC) (v1.0.4),^38^ excluding genomes with clade separation scores (CSS) > 0.45. Qualified 8,067 genomes were then retrieved and used to generate a Kraken2 database, following the identical method as above.

#### Variant calling

Variant calling with DeepVariant (v1.6.1)^39^ was performed using the WGS model with default parameters. For both calls, only variants tagged with PASS in the FILTER field were considered in downstream analysis. Variants were annotated with the pre-packaged gnomAD^51^ database v1.8.hg38.20230908g (https://console.cloud.google.com/storage/browser/broad-public-datasets/funcotator/) using Funcotator. In-house script was used to reformat Funcotator annotations into a annotation file that could be supplied to BCFtools (v1.20).^40^ VCF files were annotated, normalized, and filtered for comparison using BCFtools. The AF popmax value was used in filtering VCFs, according to GnomAD variant interpretation best practices.^52^ Variants were stratified into common (MAF ≥ 0.05) and rare (MAF < 0.05) variants.

#### Variant concordance evaluation

The performance of the decontamination was evaluated by comparing the concordance of variant calls between the truth set and either the raw or decontaminated query set. Variants identified from blood samples were regarded as the true set, and those from oral samples as the query set for comparison. Variant concordance was calculated using hap.py (v0.3.15) at the per-superlocus level, following the GA4GH best practices.^41^ Specifically, each of the stratified blood VCFs were specified as the truth VCF, and the corresponding stratified oral VCFs were specified as the query VCF. As stated in hap.py’s documentation, variants were categorized as true positives (TPs), false negatives (FNs), or false positives (FPs) based on concordance between truth and query VCFs. TPs were defined as variants present in both blood and oral VCFs. FNs were variants present in the blood VCF but absent from the oral VCF. FPs were variants detected only in the oral VCF or those with mismatched genotypes or alternate alleles.

Then, evaluation metrics of precision, recall, and F1-score were calculated using the following formula.

Precision=TP/(TP+FP)

Recall=TP/(TP+FN)

F1Score=2×Precision×Recall/(Precision+Recall)

Evaluation metrics of precision, recall, and F1-score were tested for statistical significance using the Wilcoxon signed-rank test.

#### CNV concordance evaluation

CNVs were detected using Canvas (v1.40.0).^25^ using the SmallPedigree-WGS mode for single sample germline analysis. Following recommended practice on GitHub, we filtered reference calls to extract CNV calls. F1-score, precision, and recall were computed using Truvari (v4.3.1).^42^ CNV calls were normalized with BCFtools for input into Truvari bench.

#### Analysis of recovered true variants

Recovered true variants were extracted using the isec function in BCFtools on the three VCF file sets of blood, raw oral, and decontaminated oral. The -i ‘FILTER=“PASS”' and -n∼101 options were used to get variants that are PASS variants in blood and decontaminated oral, but not the raw oral sample. These recovered true positive variants were annotated using the Variant Effect Predictor (VEP) (v113.0)^43^ using the PolyPhen_SIFT plugin. The resulting VEP output was filtered for variants annotated as “probably_damaging” in the PolyPhen_humdiv_pred field. The alignments of each blood, raw oral, and decontaminated oral sample was visualized with the Integrative Genomics Viewer (IGV) (v2.17.4).^44^

Mean precision-recall curves were generated using hap.py benchmarks on stratified regions specified by BED files, provided using the “-f” option. These BED files were downloaded from the GIAB FTP site, https://ftp-trace.ncbi.nlm.nih.gov/ReferenceSamples/giab/release/genome-stratifications/v3.0/GRCh38/. Datapoints of precision-recall were obtained using the “--roc QUAL” option. To ensure that no extrapolation occurs, only the datapoints that fall into common recall regions shared by all samples were retained and averaged. The BED files used included sequencing-resistant promoters “GRCh38_BadPromoters.bed.gz”, GC-rich genomic regions “GRCh38_gc85_slop50.bed.gz”, and functional regions “GRCh38_refseq_cds.bed.gz”.

For identification of specific microbial gene of origin for contaminant reads from HMCN2 the genomes, we obtained 1,721 conspecific genomes of *P. pasteri* (HROM_Genome_1099) from HROM. From these genomes, coding gene sequences were predicted using Prokka (v1.14.6).^45^ Predicted gene sequences were clustered using MMseqs2 (v13.45111)^32^ using “search” module with sequence identity ≥90% and coverage ≥80% to build non-redundant species pangenome of oral *P. pasteri* (HROM_Genome_1099). The contaminant reads were aligned to pangenome of *P. pasteri* using BWA-MEM. Number of reads aligned were identified using SAMtools function “coverage”. The aligned five genes were annotated using InterProScan (v5.73-104.0).^46^

#### Analysis of bacterial regions homologous to clinical variant sites

Clinical variant positions were extracted from the ClinVar (clinvar_2023071_hg38.vcf)^31^ VCF file along with their ±150 bp flanking sequences. MMseqs2 search (coverage ≥ 50%, sequence identity ≥ 50%, e-value = 1e-3) was performed using cropped sequences from clinical variant positions against 3,426 HROM species and their respective 72,641 high-quality genomes to identify matching bacterial regions. Sequences with coverage ≥ 50% and sequence identity ≥ 80% were retrieved. If the retrieved sequences were shorter than 150 bp, flanking sequences were extended to ensure a total length of 150 bp.

Single-end sequence simulation (150 bp read length, 20 × coverage) was performed using art_Illumina of ART (v2016.06.05)^53^ based on matched bacterial genomic regions. Simulated reads were aligned to the corresponding human FASTA regions using BWA-MEM. A bacterial sequence was classified as positive if ≥ 50% of simulated reads aligned to a clinically relevant human genomic region. To further validate positive bacterial contigs and remove mobile genetic elements, we used CheckV (v1.0.1)^54^ and GeNomad (v1.8.0).^55^ Contigs with a GeNomad score above 0.7 were classified as plasmids or proviral regions and subsequently removed. Additionally, any contigs classified as “High-quality” by checkv quality were excluded. In total, seven contigs were removed. Finally, we used FCS-GX^47^ to remove the matched contigs that are deemed as contaminants from *Homo sapiens*.

### Quantification and statistical analysis

Statistical analyses were performed in R (v4.4), with details of tests and parameters provided in the Results, Figure Legends, and STAR Methods. Data visualization was carried out using ggplot2 (v3.5.2)^48^ and ggsignif (v0.6.4).^49^ The *p*-values for comparisons of decontaminated read counts were calculated using the Mann-Whitney U test, while the *p*-values for comparisons of variant concordance were carried out using the Wilcoxon signed-rank test. *p*-values are shown with asterisks denoted as ∗, *p* < 0.05; ∗∗, *p* < 0.01; ∗∗∗, *p* < 0.001; ∗∗∗∗, *p* < 0.0001.

## Supplemental information

Document S1. Figures S1–S5

Table S1. Decontamination results for whole blood samples, related to Figure 1

Table S2. Summary of variant calling by DeepVariant, related to Figure 2
