# Supplementary material for: Metagenome-assembled genomes enhance bacterial read decontamination and variant calling in oral samples
Source: iScience. 2025 Oct 14;28(11):113772. doi: 10.1016/j.isci.2025.113772 (PMC12616088; doi:10.1016/j.isci.2025.113772)
Supplement: Document S1. Figures S1–S5 [file mmc1.pdf]

iScience, Volume *28*

Supplemental information

Metagenome-assembled genomes enhance bacterial read decontamination and variant calling in oral samples

Zunu An, Jun Hyung Cha, Kyu Ha Lee, and Insuk Lee

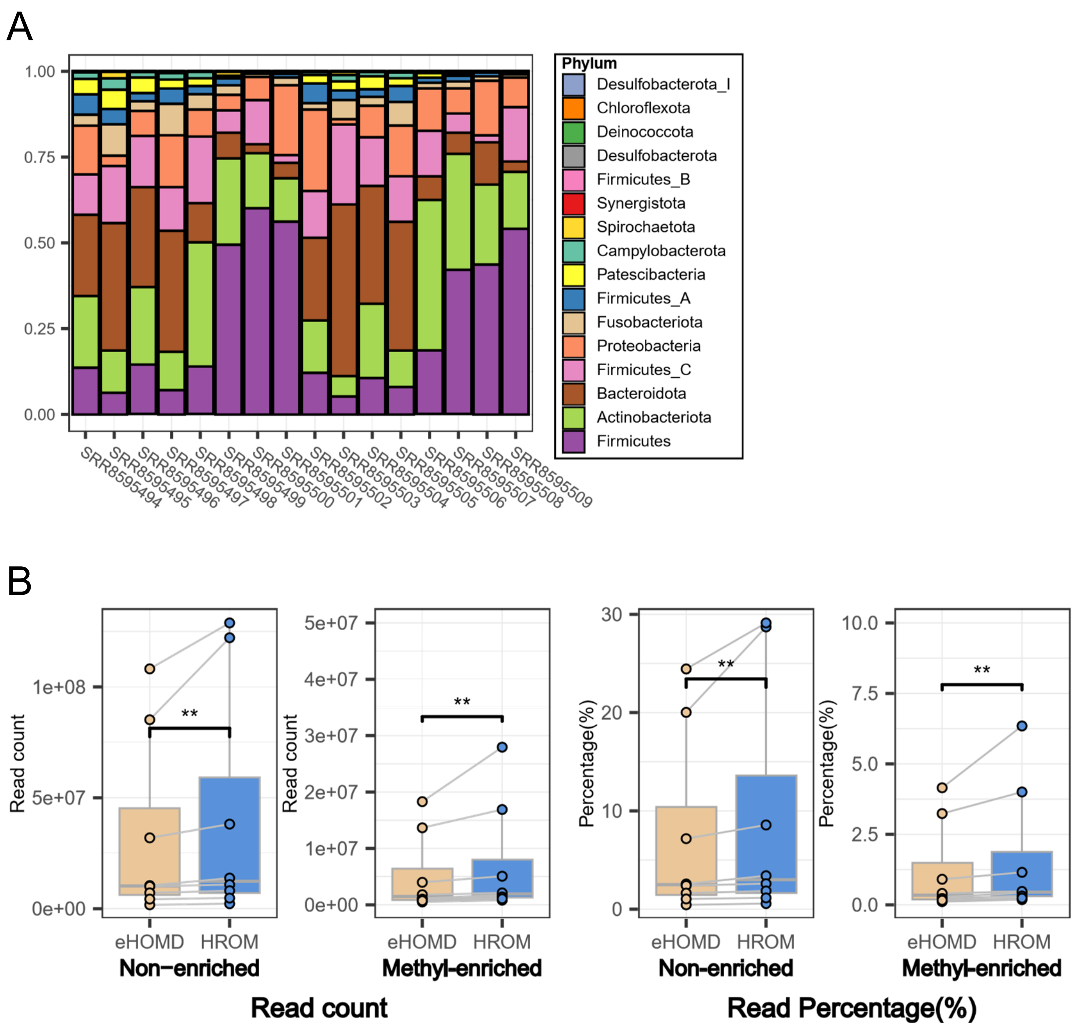

Figure S1. HROM outperforms eHOMD in detection of contaminated bacterial reads, related to Figure 1. (A) Bar plot represents phylum composition of oral bacterial contaminants per sample measured by HROM. (B) Box plots on the left and right show the read count and percentage of identified contaminants for each database. Statistical significance was assessed using a one-tailed Wilcoxon signed-rank test. Box plot elements include the median (center line), interquartile range (box edges at 25th and 75th percentiles), and whiskers extending to 1.5× the interquartile range.

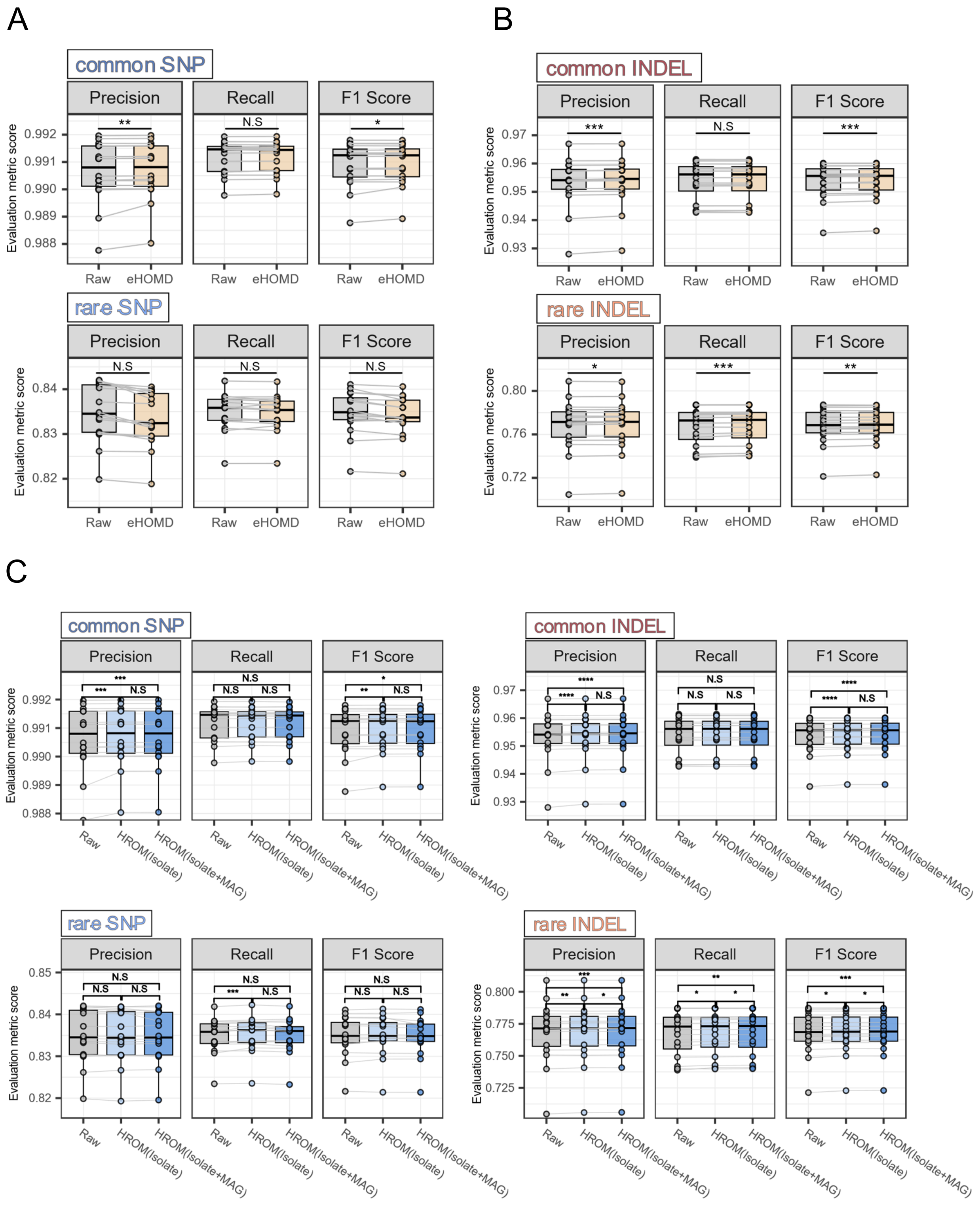

Figure S2. Raw metric scores for variant concordance, related to Figure 2. (A-B) Box plots show the distribution of precision, recall, and F1 scores for common (MAF ≥ 0.05) and rare (MAF < 0.05) SNPs (A) and indels (B), comparing eHOMD-based decontamination (light orange) with raw sequencing samples (light gray). (C) Box plots show the distribution of precision, recall, and F1 scores for common (MAF ≥ 0.05) and rare (MAF < 0.05) SNPs and Indels using three contaminant filtering strategies: raw (no decontamination), isolate-only HROM, and entire HROM. Boxplot elements consist of the median (center line), interquartile range (box edges at 25th and 75th percentiles), and whiskers extending to 1.5× the interquartile range.

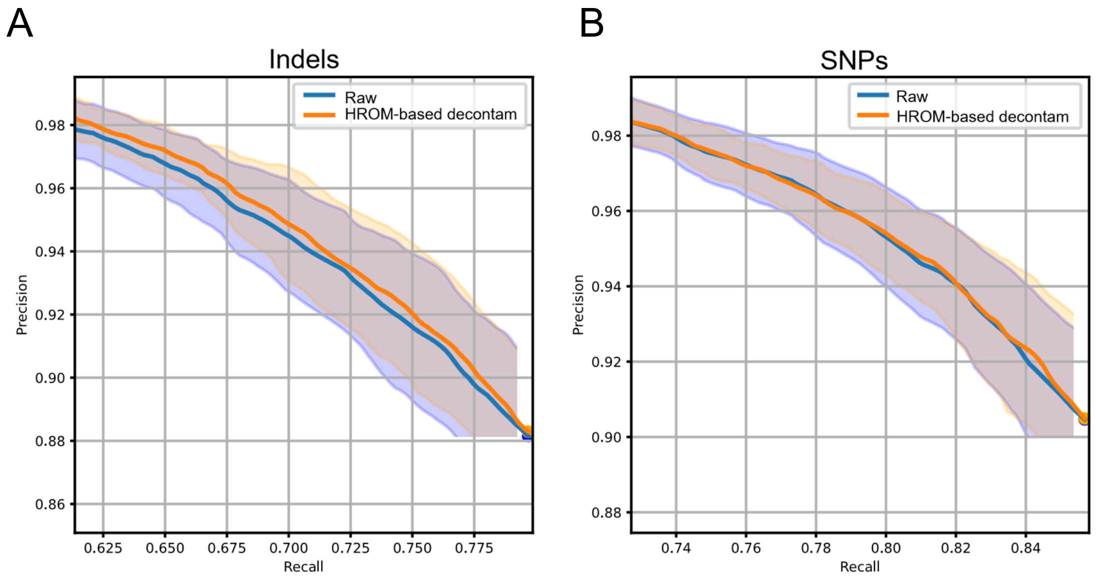

Figure S3. Decontamination of bacterial reads enhances recovery of variants particularly in GC-rich regions, related to Figure 3. Mean precision-recall curve comparing HROM-based decontamination (orange) and raw (blue) in GC-rich regions (GC-content > 85%) for Indels (A) and SNPs (B). Shaded regions for both plot represent one standard deviation, with overlapping areas between methods shown in gray.

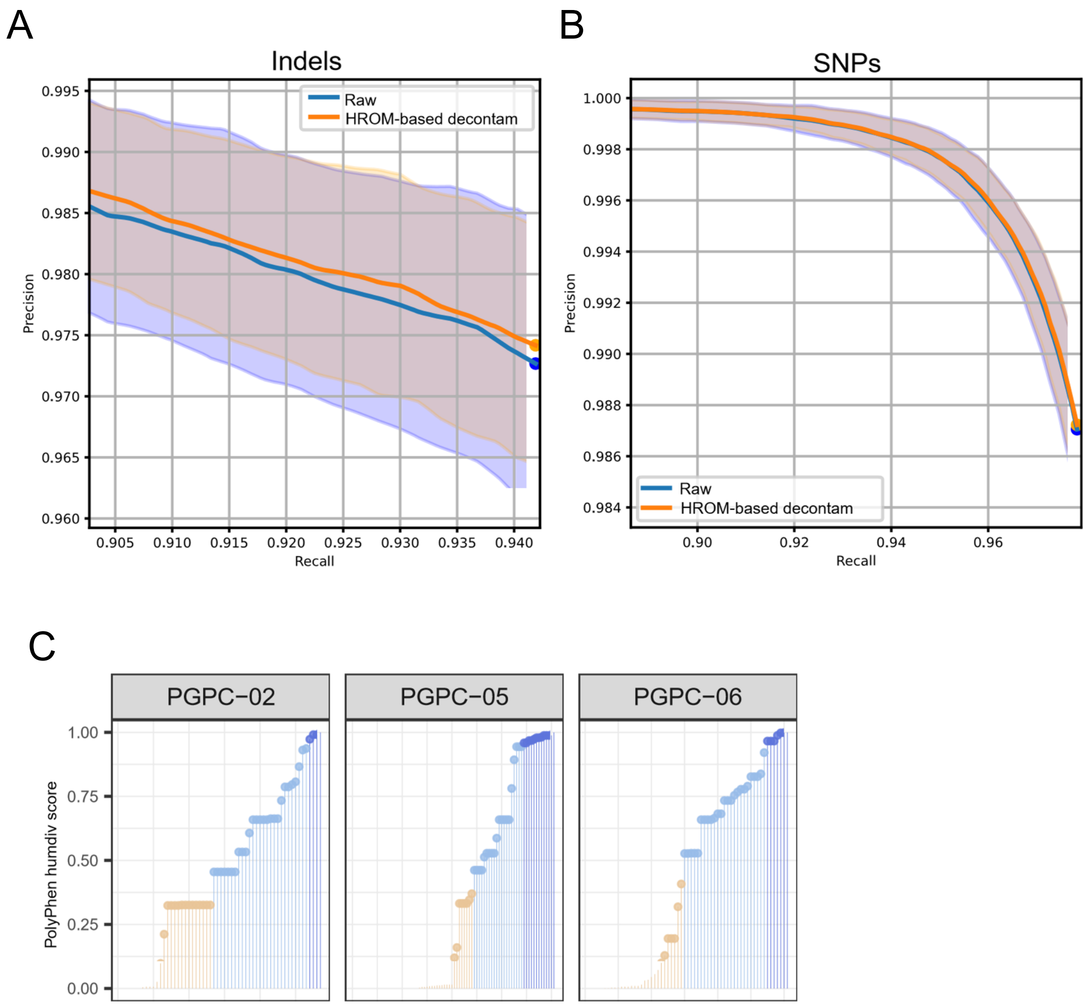

Figure S4. Recovery of true variants through HROM-based decontamination of bacterial reads, related to Figure 4. Mean precision-recall curve comparing variant calling results from samples with HROMbased bacterial read decontamination (orange) and raw samples (blue) in coding regions (GC-content > 85%) for indels (A) and for SNPs (B). Shaded regions for both plots represent one standard deviation, with overlapping areas between methods shown in gray. (C) Recovered missense variants sorted by ascending PolyPhen-2 scores for individuals PGPC-02, PGPC-05, and PGPC-06, supplementing results for PGPC-50 in Fig. 4b. Variants are color coded according to its predicted functional effect of benign (yellow), possibly damaging (light blue), and probably damaging (dark blue).

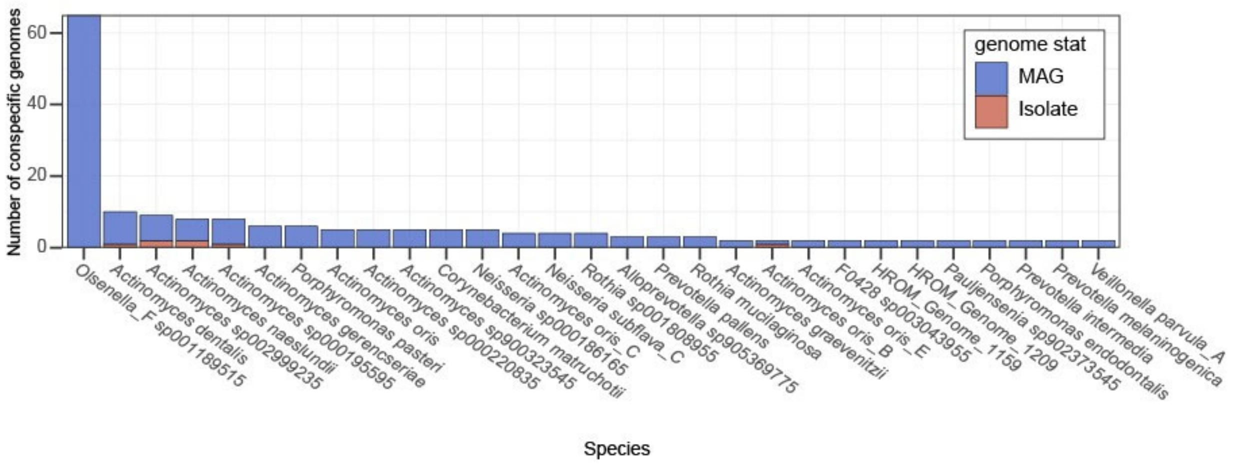

Figure S5. Alignment in ClinVar variant regions and number of matching conspecific genomes across species, related to Figure 5. Each bar represents the number of genomes per species in HROM that contain contigs with high sequence similarity (≥50% coverage and ≥80% identity) to human genomic regions flanking ClinVar variants. Bar colors indicate whether the contigs originated from MAGs (blue) or isolate genomes (red).
